# Detection of specific chromosomal aberrations in urine using BCA-1 (oligo-CGH-array) enhances diagnostic sensitivity and predicts the aggressiveness of non-muscle-invasive bladder transitional cell carcinoma

**DOI:** 10.1007/s00345-013-1191-3

**Published:** 2013-11-07

**Authors:** Olivier Cussenot, Karim Sighar, Mansoor Mohammed, Sylvain Hugonin, Valérie Ondet, Stéphane Larre, Roger Lacave, Morgan Roupret, Géraldine Cancel-Tassin

**Affiliations:** 1 CeRePP, Paris, France; 2 GRC-05, Institut Universitaire de Cancerologie (IUC), University Paris-6, Paris, France; 3 Department of Urology Tenon and Pitié Hospitals, University Paris-6, Paris, France; 4 Departments of Tumor Biology, Tenon Hospital, IUC, University Paris-6, Paris, France; 5 ArrayGenomics, Voisins-le-Bretonneux, France; 6 Managenedx, 1277 Kestell Blvd, Oakville, ON L6H0B3 Canada

**Keywords:** Transitional cell carcinoma, Bladder, Cytology, CGH-arrays, Cytogenetics, Markers

## Abstract

**Introduction:**

Bladder carcinoma (B-TCC) is the fifth most prevalent carcinoma in the United States (US) or Europe. In addition, B-TCC is the most expensive carcinoma per patient between diagnosis and death, because of its 50–80 % recurrence rate. B-TCC is an optimal carcinoma for which to detect DNA alterations in urine, which is easily obtainable. Chromosomal aberrations in tumors have been closely related to the carcinogenesis process.

**Material and Methods:**

We developed a highly specific and sensitive oligo-CGH-array for the diagnosis and follow-up of B-TCC, based on the detection of chromosomal aberrations in urine samples. One hundred and sixty-four urine samples were analyzed. The qualitative results, including chromosomal aberrations, were obtained. Quantitative results are expressed as a percentage of chromosomal alterations on the autosomes.

**Results:**

From the urine samples, we were able to differentiate B-TCC from non-malignant conditions with an accuracy of 100 % for patients without history of B-TCC. For follow-up of B-TCC in clinical practice, at least a deletion (8p; 9p; 9q) or a cut-off of >2 % of chromosomal imbalance was considered as a positive test. According to our criteria, 100 % of high-grade tumors were diagnosed, and the sensitivity to predict positive cystoscopy was 95 % (specificity 73 %). A cut-off >9 % was a strong signature of high-grade TCC (odds ratio 53 CI 95 % 7–417; *p* = 0.0002).

**Conclusion:**

We developed a sensitive clinical tool for the detection of B-TCC using DNA extracted from patient urine. This tool is also able to identify low-grade B-TCC and identify high-risk patients harboring a high-grade disease.

## Introduction

Bladder transitional cell carcinoma (B-TCC) is the fifth to seventh most prevalent carcinoma in the United States (US) or Europe. In the USA, 69,250 new cases of bladder cancer were diagnosed and an estimated 14,990 deaths occurred in 2011. Non-muscle-invasive B-TCC has the highest recurrence rate of any carcinoma. In addition, B-TCC is the most expensive carcinoma per patient between diagnosis and death, because of its 50–80 % recurrence rate. Moreover, progression from a high-grade non-muscle-invasive cancer to a muscle-invasive cancer occurs in 10–20 % of cases. In addition, bladder carcinoma is the most expensive carcinoma per patient between diagnosis and death because of its 50–80 % recurrence rate [1].

The current standard for diagnosing B-TCC that includes the cytological examination of cells present in voided urine alone does not provide a safe screening alternative for cystoscopy because of its low sensitivity (<30 %), especially for the detection of low-grade tumors. Consequently, the standard procedure for diagnosing bladder carcinoma recurrence is flexible cystoscopy. However, as the sensitivity of cystoscopy is approximately 80 %, some tumors could escape detection, especially in the case of flat tumors (Tis), justifying the concomitant use of cytology and the development of fluorescence cystoscopy [2]. Stenzl et al. [3] reported that the percentage of lesions missed by standard light cystoscopy but detected using fluorescent cystoscopy ranged between 10 % (high grade) and 55 % (atypia). Moreover, in approximately 10 % of cases, B-TCC could be associated with upper urinary tract tumors, justifying a follow-up using CT-urography, especially when the cytology is positive and the cystoscopy is negative. Cystoscopy is an invasive diagnostic approach that is unpleasant for the patient, with iatrogenic risks such as infections. Consequently, the actual practice of surveillance for patients with superficial bladder cancer differs substantially from the standards recommended in clinical guidelines [4, 5]. After resection of the primary tumor, guidelines advocate that patients undergo such procedures every 3–12 months for at least 5 years in the case of low-risk disease and for life in the case of high risk disease [6]. A number of noninvasive tests to detect urinary non-muscle-invasive B-TCC have been developed in order to overcome the low sensitivity of cytology and to reduce the number of irrelevant cystoscopy. Current urinary biomarkers for the detection of non-muscle-invasive B-TCC have been reviewed by Tomasini et al. [7]. ELISA tests (BTA stat, BTA TRAK and NMP-22) and cytology-based tests (ImmunoCyt/uCyt™ and UroVysion™) have obtained either Food and Drug Administration (FDA) clearance or approval [7]. Several reports have suggested the diagnostic utility of genetic (and epigenetic) markers [8]. Since the work of Sidranskyet al. [9, 10] in 1996, the detection of genomic or mitochondrial DNA alterations has been able to provide early detection of B-TCC with a high sensitivity (>70 %) and specificity (>70 %). Moreover, a better understanding of the molecular pathways [10–12] involved in bladder carcinogenesis has led to the development of translational molecular analyses [loss of heterozygosity (LOH), hypermethylation of CpG-islands, point mutations] for diagnostic or prognostic purposes, from DNA isolated from cells present in voided urine samples. Aberrations on the 9p, 9q and 8p chromosomal arms are the most common events identified in non-muscle-invasive B-TCC using LOH analysis or CGH-arrays [13]. Currently, it is generally recognized that FGFR3 gene mutations and low chromosomal instability are associated with non-aggressive superficial tumors and that high chromosomal instability and TP53 mutations drive progression to invasive carcinoma. Commercial tests have been developed based on cells present in voided urine samples or the analysis of their DNA. The FDA-approved test (Urovysion™) is based on the detection of aneuploidy for chromosomes 3, 7, and 17 and loss of the 9p21 locus. We and others have previously shown that the detection of recurrent bladder cancer can be improved by DNA analysis (LOH and DNA CpG-island hypermethylation) from voided urine. More recently, we have developed a specific and inexpensive CGH-array [14] that combines diagnostic and prognostic performance for use in clinical practice. The array covers, with a high density, the target loci reported to be frequently gained or deleted in bladder carcinoma and, with a low density, the entire genome. The aim of this study was to measure the diagnostic performance of this urinary marker test to predict positive cystoscopy during the follow-up of non-invasive B-TCC and to assess its performance in order to identify patients with a risk of high-grade disease.

## Materials and methods

### Study design

#### Patients and sample collection

In a first group, thirty patients were enrolled as controls (no symptoms of bladder carcinoma, no history of urologic carcinoma), and they were all explored by cystoscopy for low urinary tract symptoms related to benign urologic conditions: 15 women for incontinence (55–75 years old) and 15 men for benign prostatic hyperplasia (50–80 years old). BCA-1 and cystoscopy were performed on second group of 134 patients during their usual follow-up for a history of non-invasive transitional cell carcinoma of the bladder. Patients with positive cytology were excluded because the main application of BCA-1 was to identify high-risk patients with false-negative cytology. All positive cystoscopy was completed by biopsy on suspect lesions for pathological analysis. All of the patients had a computed tomography urography for <2 years in order to explore upper urinary tract. The follow-up of patient after BCA-1 was at least one year for all patients. Urine samples were collected during patient visits or in the morning before any surgical procedure. Urine samples were collected for all patients using DNA preservation medium (Norgen™) in case of office-based outpatient setting. Urine was spun at 350 g (1,500 rpm) for 10 min and washed twice with phosphate-buffered saline and stored at −80 °C. DNA was extracted from pellets using the QIAamp DNA Blood Mini Kit (Qiagen, Hilden, Germany) according to manufacturer’s instructions. Study participants provided written informed consent, and the study protocol received approval from the institutional review board.

#### Comparative genomic hybridization array design

CGH was performed using the BCA–oligo CGH-array (ArrayGenomics, Voisins-le-Bretonneux, France). This array consists of a glass slide spotted with 60,000 oligonucleotides (oligo) covering genomic areas known to undergo modification in TCC of the urinary tract. The oligonucleotide is a 60-mer oligonucleotide which represents, in all, 56.7 Mb (2 % of the genome). The microarrays are manufactured using a proprietary non-contact industrial inkjet printing process, in which oligo-monomers are deposited uniformly onto specially prepared glass slides. This in situ synthesis process prints 60-mer-length oligonucleotide probes, base by base, from digital sequence files. The precise inkjet process enables the delivery of extremely small, accurate volumes of the chemicals to be spotted. This technology, SurePrint, allows as many as 8 arrays to reside on a single slide (8 × 60,000).

#### Labeling

A total of 200 ng of urinary DNA was labeled by random priming with Cy5, and 200 ng of normal genomic DNA (Promega, Madison, WI, USA) was marked with Cy3 using the complete labeling kit BioPrime Total Genomic Labeling System (Invitrogen, Carlsbad, CA). Excess labeling was eliminated using the BioPrime Purification Module with Purelink (Invitrogen, Carlsbad, CA).

After labeling, the urinary DNA and the normal DNA were combined with Cot-1 DNA (Invitrogen, Carlsbad, CA), Agilent 10X Blocking Agent and Agilent 2X Hybridization Buffer. This mix was then incubated first at 95 °C for 3 min and then immediately at 37 °C for 30 min.

The labeled DNA was deposited on the gasket slide, on which the BAC–oligo spotted slide was then placed. The hybridization was performed at 65 °C for 18 h. The BCA–oligo slide was washed with two solutions: wash buffer 1 for 5 min at room temperature and wash buffer 2 for 1 min at 37 °C (Agilent Technologies). The slide was then dried by centrifugation.

### Scanning and data analysis:

The BCA–oligo array was scanned at a 3 micron resolution on the high-resolution Agilent scanner. The scanner measures the fluorescence intensity of two dyes simultaneously (wavelengths of 532 and 635 nm) from labeled samples of nucleic acid bound to the BCA-1 test.

The scanned images were analyzed using Feature Extraction software, and the graphic display of the data was generated by Cytogenomics software (Agilent Technologies). Each oligo was positioned in the human genome in accordance with NCBI Build 37 (UCSC hg19, February 2009). Cytogenomics software uses the Agilent ADM-2 algorithm. For these analyses, a probe was deemed to be deleted if its standardized log2 ratio was <−0.2 and was duplicated if its standardized log2 ratio was greater than 0.2.

**Fig. 1:**
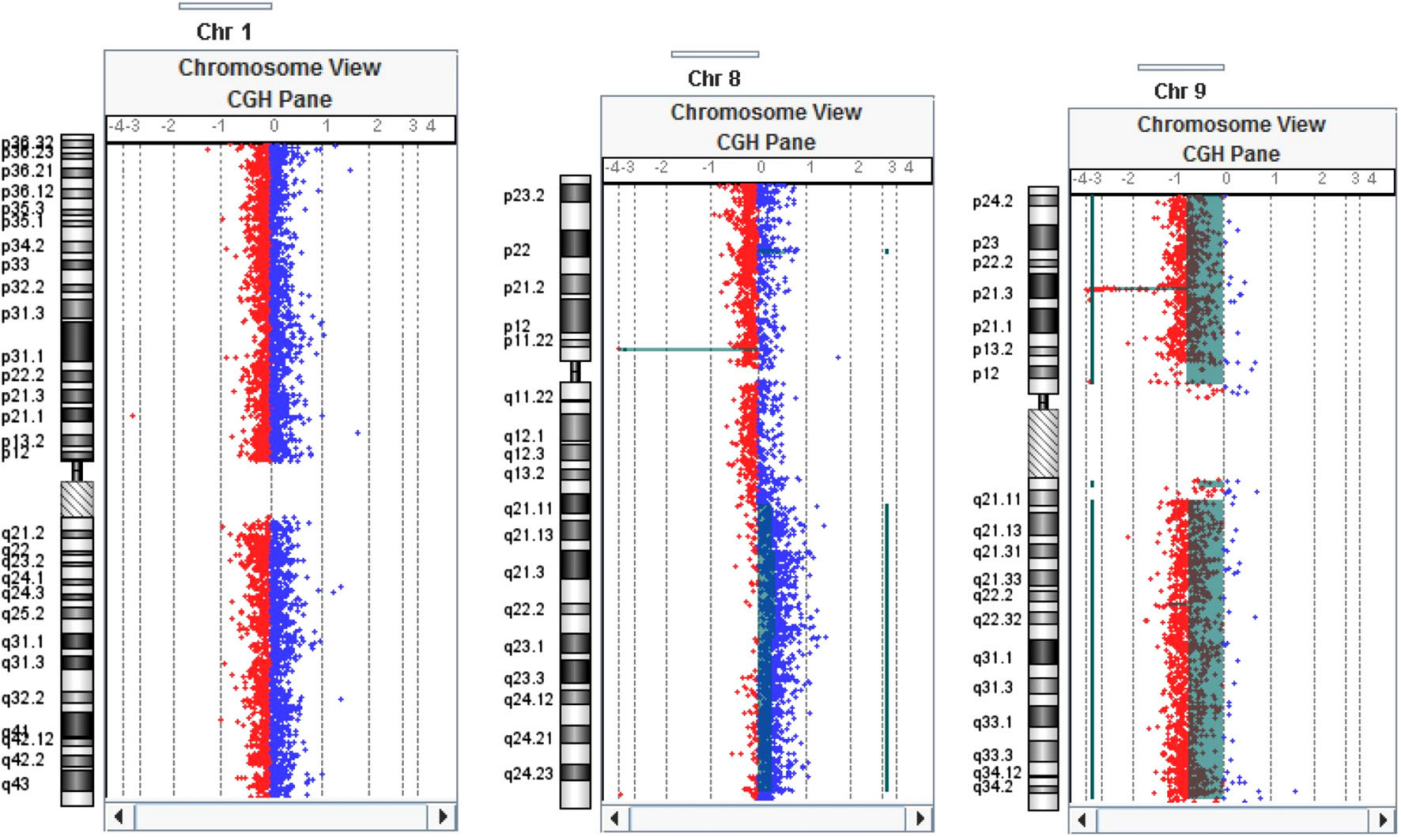
Examples of normal profiles on chromosome 1, a gain on the 8q chromosome arm and a deletion on the 9p chromosome arm at the 9p21 locus

The software segments displayed the data by grouping together those probes which have a proximate position in the genome and ratio averages which correspond to loss or gain (Fig. 1).

#### Statistical analysis

**Fig. 2:**
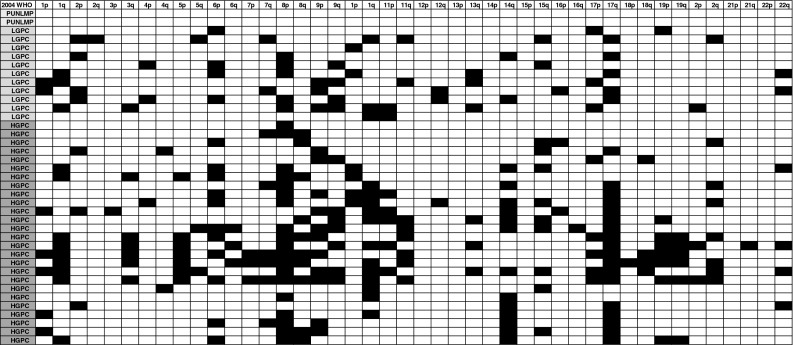
Synoptic table of chromosomal arms by aberrations according to the 2004 WHO aggressiveness classification of non-muscle-invasive B-TCC. *PUNLMP* papillary urothelial neoplasm of low malignant potential, *LGPC* and *HGPC* low-grade and high-grade papillary carcinoma

In the first group of controls, patients were analyzed individually for chromosomal aberrations. In the cohort of patients with history of B-TCC, accuracy, sensitivity, specificity and predictive values were calculated using as endpoint the diagnosis of bladder tumor at cystoscopy (qualified as positive). According to our previous model (Larre et al.), the BCA-1 oligo array was defined as positive if at least one chromosomal arm aberration was observed for the following loci: deletions (8p, 9p, 9q) or if the percentage of aberrations on the 22 autosomes was >2 %. The secondary endpoint was the aggressiveness (2004 WHO classification) of the tumor on pathological examination of the biopsy of the tumor. A high risk of high grade (G3) for the biopsy sample was defined as positive if the percentage of aberrations on the 22 autosomes was >9 %. A synoptic table of chromosomal arms by aberrations is given in Fig. 2, according to the 2004 WHO aggressiveness classification of non-muscle-invasive B-TCC. We looked for Pearson correlation between chromosomal aberrations given in Fig. 2. Finally, BCA-1 positivity (>9 %) was calculated for presence and absence of high-grade tumor on the biopsy. The distributions were compared by logistic regression. Odds ratios (ORs) as a measure of relative risk of high-grade disease and their 95 % confidence intervals (CIs) were estimated. Software MedCalc12.7.0 (Acacialaan 22, B-8400 Ostend, Belgium) was used for statistical analysis.

## Results

Details of positive BCA-1 test were given in Table 1 according to history of urothelial tumors, result of cystoscopy and result of histopathology on biopsy. First of all, we evaluated percentage of the false positive on the control group (patients without history of B-TCC). Any chromosomal aberrations (any false positive) were identified in the totality 30 control patients giving an accuracy of 100 %.

**Table 1:** Details of positive BCA-1 test according to history of urothelial tumors, result of cystoscopy and result of histopathology on biopsy Pearson correlation coefficients between chromosomal arm aberrations at 8p, chromosome 9 and chromosome 17

	N	BAC-1 +(>2 % chromosomal aberrations) for the prediction of positive cystoscopy (recurrence)	BAC-1 +(>9 % chromosomal aberrations) for the prediction of High-grade tumor on biopsy
Controls: No history of Urothelial tumors Negative cystoscopy	30	0	0
Cases: History of Urothelial tumors	134		
Negative cystoscopy	95	46	20
Positive Biopsy	39	37	34
Details according histopathology			
PUNLMP	2	0	0
LGPC	11	11	8
HGPC	26	26	26

Secondly, we evaluated the accuracy (64 %), sensitivity (95 %), specificity (51 %), positive predictive value (45 % CI 95 % 34–55) and negative predictive value (96 % CI 95 % 86–98) of BCA-1 test considering positive cystoscopy as endpoint. Aberrations on 9p/q and 8p were not correlated together but correlated significantly with 17p/q aberrations [Pearson correlation coefficient (*p* value) was respectively: 0.26 (*p* = 0.0017); 0.35 (*p* < 0.0001); 0.65 (*p* < 0.0001) between locus 8p and 9p/q; locus 8p and 17p/q; locus 9p/q and 17p/q].

Aberrations on 9p/q, 8p, combined aberrations at 8p or 9p/q and a ratio of >2 % of chromosomal arm aberrations were significantly associated with positive endoscopy with an odds ratio and p value given in Table 2.

**Table 2:** Odds ratios (logistic regression) for 8p- or 9p-, 9q-, 8p- or 9p- or 9q-, ratio >2 %

Dependent variable: positive cystoscopy	Odds ratio	95 % confidence intervals	*p* value
8p	8.7	3.3–23.1	<0.0001
9p	4.8	1.1–21.1	=0.0358
9q	15.1	1.5–152.2	=0.0207
8p or 9p/q	8.0	2.8–22.3	=0.0001
Ratio >2 %	5.7	1.1–29.3	=0.0367

Thirdly, we looked for the relationship between the diagnosis of high-grade disease on biopsy and the chromosomal aberrations observed. The percentage of alterations increased with the grade/stage of the tumor (Fig. 2). A percentage value of >9 % for chromosomal arm aberrations on the 22 autosomes was associated with a high risk of high-grade on histological examination of the biopsy samples with an odds ratio of 53 (CI 95 % 7,417 *p* = 0.0002). According to our criteria, 100 % of high-grade tumors were diagnosed, and the sensitivity, specificity, accuracy, positive predictive value and negative predictive value were, respectively, 100, 75, 78 %; (39 % CI 95 % 27–53) and (100 % CI 95 % 95–100).

## Discussion

DNA alterations and cytogenetic aberrations have been recognized to be closely linked to the natural history of B-TCC. BCA-1 test is a robust assay based on DNA analysis of voided urines allowing to identify the main chromosomal aberrations related to B-TCC natural history. We show that BCA-1 has no false positive, in population of patients with normal cystoscopy, normal cytology and no history of B-TCC. BCA-1 detects all patients with a high-grade disease recurrence of B-TCC and has a sensitivity of 95 % for the prediction of a positive cystoscopy for patients followed for a history of B-TCC. The non-invasive detection and monitoring of B-TCC recurrence remain a challenge to reduce non-relevant invasive urinary exploration using iterative cystoscopy and to replace voided urinary cytology, which suffers from low sensitivity (25–40 %) and observer-dependent variability. The detection of chromosomal instability at a specific locus has previously been demonstrated, by us and others, to show a high sensitivity (over 70 %) and specificity (over 70 %) using multiplex microsatellite analysis or by CGH/SNP-arrays [14, 15]. Imbalances on chromosomes 8p and 9p/q have been reported as the most common events in non-muscle-invasive B-TCC. We confirmed the observation of Bulashevska et al. [16] who showed that 8p and 9p/9q imbalances are mutually exclusive at early stages but correlate together with 17p/q chromosomal aberrations during progression. Chromosomes 8p and 9p/q aberrations are identified in 78 % of tumor recurrences, including 100 % of high-grade disease cases. Only low-grade diseases harbor rare and heterogeneous chromosomal imbalances. Two papillary urothelial neoplasm of low malignant potentials (PUNLMPs) were found to have any chromosomal aberrations on BCA-1, and this observation has been previously reported by Chow et al. [17].

As false positives were not present in the control population without a history of B-TCC, patients with a history of B-TCC “false positives” (36 % in our hand) could be possible false negatives of cystoscopy (10–55 % from high grade to atypia reported in the literature). Moreover, according to the previous observations with microsatellite analysis, positive cystoscopy could be postponed for 6–24 months after the detection of specific LOH in urine [9].The commercially available FDA-approved Urovysion™ test is also based on the detection of aneuploidy (centromeric markers) for chromosomes 3, 7, and 17 and the loss of the 9p21 locus, using multiprobe FISH, on cells obtained from voided urine [18]. Urovysion™ test gives an intermediate sensitivity of <70 % because it is limited to 2 endpoints: aneuploidy (using centromeric markers) and the bladder-specific deletion at 9p21 locus, which occurs in only approximately 25 % of cases. This fact has been clearly demonstrated by Panzeri et al. [19]. Thanks to the comparison of chromosomal aberrations in bladder cancer by targeted FISH versus wide microarray-based CGH analysis. Moreover, Urovysion™ needs to be coupled to cytopathological examination, which is also time-consuming and has observer-dependent variability. Deletion at chromosome 10q or gains on chromosomes 8q, 17q and 20q have been associated with progression using CGH analysis of tissue [15, 20]. Moreover, chromosomal instability, quantified using the percentage of copy changes, has been shown to be related to high-grade and invasive tumors [15]. In our hands, the number (percentage) of chromosomal aberrations (over 9 %) is a strong marker for determining which patients are at a high risk of having a high-grade disease.

Other reports suggest that point mutation analysis of the FGFR3, RAS and TP53 genes could be used on urine-voided DNA to diagnose recurrent tumors [21, 22]. FGFR3 mutations are associated with recurrent, chromosome-stable and low-grade tumors, and they are mutually exclusive from RAS and TP53 mutations associated with chromosome-unstable and high-grade tumors. The prevalence of the more frequent mutations in exon 7 and exon 10 of the FGFR3 gene [23] remains relatively low (<30 % for low-grade B-TCC) and could be used to monitor follow-up when a specific mutation has been previously identified in the primary tumor.

The concept of the presence or absence of one or a small number of molecular markers in a relevant diagnostic or prognostic test in oncology contrasts with the heterogeneity of solid tumors such as bladder carcinoma. However, at this time, multiplex signatures are commercially available for clinical practice.

## Conclusion

High-throughput CGH-arrays [14], single-nucleotide polymorphism arrays [15] or sequencing microarrays for mitochondrial mutation detection [24] have demonstrated their strong suitability for diagnostic/prognostic applications but remain too expensive for clinical practice. BAC-1 is the first commercially available microarray based on CGH analysis that has been developed for clinical practice (with the possibility to screen 8 patients on the same chip) and could be used in an office-based outpatient setting.

Future trials using BAC-1 on a larger cohort and comparison of our results to other bladder tumor marker assays may help us to identify the best and the most cost-effective strategy to decrease discomfort and allow for more accurate follow-up of patients with non-invasive B-TCC.
